# Chloroform in Obstetrical Practice

**Published:** 1875-09

**Authors:** Daniel Barry

**Affiliations:** Flatwoods, Tennessee


### CHLOROFORM IN OBSTETRICAL PRACTICE.

By DANIEL BARRY, M.D., Flatwoods, Tennessee.

*Editors Atlanta Medical and Surgical Journal:*

In your July number, 1875, I notice a discussion had by the members of the Atlanta Academy of Medicine, some in favor of and some against the use of anæsthetics in obstetrical cases.

Considering it the duty of every practitioner to record his views and experience on subjects of importance, I have decided to venture an article for your Journal.

In surgical practice, I have for a good many years been in the habit of using as an anæsthetic an article which I call chloric ether, consisting of a mixture one-quarter chloroform and three-quarters ether, with perfect satisfaction. Pure or unmixed chloroform I never use if I can help it, as I regard it as extremely dangerous. I took a big scare at chloroform (used alone) in the outset of my professional career, and have never regained sufficient confidence in it to use it alone, excepting with such extreme caution as to amount to timidity. But I consider the combination given above as almost entirely free from danger in any quantity necessary to produce the desired result, and as more certain and rapid in its action than ether alone.

In obstetrical practice, however, I am opposed to the use of anæsthetics *in toto*, unless in cases where it becomes necessary to perform some painful or dangerous operation or manipulation. Parturition I look upon as a natural process, and the pains of labor as natural pains, and am opposed to interference by the accoucheur in any manner, under ordinary circumstances.

For hundreds of years before the discovery of anæsthetic agents, women bore the pains of labor, and no worse results (if, indeed, as bad results) followed than since their introduction into use in these cases. I can think of but two reasons which prompt the medical man to use agents of this class in midwifery cases. One of these is a dread of hearing the cries and complaints of the woman in travail, and the other to be in the *fashion*, to humor the whims of ladies who are too delicate, *i.e.*, too aristocratic and fashionable, to do as their mothers did, and bear the pains of maternity with the greatest degree of fortitude of which they are capable. Seriously, I think that the woman who will risk the dangers of inhaling chloroform under the circumstances, after these dangers are fully explained to her by her medical attendant, is criminally foolish, to say the least of it; and the man who by authority writes M.D. after his name, who will administer any agent of this class to a parturient woman without fully and fairly explaining all the danger from every source which attends its administration in such cases, should be indicted for malpractice if any bad consequences follow. Any practitioner may allow himself to be persuaded to do what his judgment does not approve; but when he does yield to urgent entreaty under such circumstances, it should always be after a full and fair explanation of the danger, and then under protest. I am no alarmist, and I am a strong believer in scientific progress but this looks to me like a step in progress in the wrong direction.

The pains of the parturient effort I will admit are sometimes terrible, but I have yet to learn that they ever kill. They are natural to the reproductive effort, and subside naturally when this effort is over. They are in some degree necessary, to old fogies like your present correspondent, as indicating very often the advancement of the labor. For all these reasons I think it right to let well enough alone. Let nature have her way, unless some malposition of the child, or some other cause, calls for the intervention of the accoucheur, and then if it be necessary to perform some extremely painful or dangerous manipulation or operation, let the whole matter be fully explained to the patient and friends; and by all means save the patient from as much artificial pain as possible. The danger from the use of chloroform in these cases consists, I think, in the largely increased probability of post partum hemorrhage. I have not, in a period of twenty-five years, and with no inconsiderable number of midwifery cases, had but four dangerous floodings to combat.

Of these one was from complete inversion of the uterus, and the other three were in patients to whom I had administered chloric ether; and I will remark just here that these three cases cured me of any desire to be a fashionable doctor. I am content to be considered "almost a heathen," and not run any more risk in any such cases.

Besides this omnipresent danger, great inconvenience results from the use of these agents, by the suspension of the action of the muscles of voluntary motion. That suspension of this action is inevitable when the anæsthetic is pushed to the point of relieving the patient of pain, I presume no one will deny; and less than that point is not only useless but absolutely nonsensical.

Nervous and muscular prostration, in some degree, as surely follows anæsthesia as night follows day; hence the danger of post partum hemorrhage from the loss of contractile power in the various muscular fibres of the parts concerned in the process of delivery, and the mouths of the uterine sinuses are left exposed and relaxed, liable to give exit to blood in dangerous, if not fatal, quantity.

These are my reasons, hastily and imperfectly given, for being "almost a heathen."

---

THE MONASTERY OF ALTENBERG have advertised for a resident physician, who is to receive, besides his board and lodging, a salary of about $100 per annum. For this munificent salary he is not only to give his professional services to the sick in the monastery, but will be expected to take charge of the hair and beards of the holy brethren.—*Medical Record.*

